# Bilateral Variations of Pronator Teres in the Upper Limb

**DOI:** 10.7759/cureus.66996

**Published:** 2024-08-16

**Authors:** Mathangi Rajaram-Gilkes, Kristi Fung, Calvin Kiniale, William Adams

**Affiliations:** 1 Medical Education, Geisinger Commonwealth School of Medicine, Scranton, USA

**Keywords:** abnormal origin of pronator teres, brachial artery absence in cubital fossa, median nerve absence in cubital fossa, variation of pronator teres, abnormal cubital fossa contents

## Abstract

Introduction: During clinical practice, physicians need to have a sound knowledge of vascular and nerve variations in the body. Patients presenting with various clinical signs and symptoms need to be thoroughly investigated with anatomic variations in mind to prevent misdiagnosis. Most nerve variations are related to their formation or their course and are frequently associated with the variability of structures that surround them. These structures most commonly include blood vessels, ligaments, and muscles. Such variations should be foremost in a physician’s mind when analyzing clinical symptoms. This will aid in accurate diagnosis, and if surgical intervention is warranted, such awareness would minimize intraoperative errors. This article discusses a variation in the pronator teres muscle, the branching pattern of the brachial artery, and the median nerve.

Materials and methods: During the dissection of 11 cadaveric specimens within the Geisinger Commonwealth School of Medicine, an elderly female cadaver exhibited bilateral variations in the pronator teres muscle, which originated from the mid-humerus, instead of the medial epicondyle. Careful dissection revealed associated neurovascular variations in the arm, elbow, and forearm in relation to the muscle. The pronator teres muscles in the remaining 10 cadavers in the lab were examined for variations and their lengths were measured and compared with the cadaver under study.

Result: Unlike the normal origin at the medial epicondyle as described in textbooks, it was observed that the humeral heads of the pronator teres muscle originated at mid-humerus level bilaterally, associated with the passage of the median nerve and ulnar artery posterior to it. This muscle was 19 cm in length bilaterally, approximately 5.5 cm longer than the average lengths of pronator teres measured bilaterally in the other cadavers. The abnormally high origin of this muscle was associated with the finding of a median nerve coursing posterior to it to the forearm, failing to appear in the antecubital fossa. Although the ulnar head appeared normal, there were bilateral variations in the median nerve during its passage between the two heads of the pronator teres at the proximal forearm as it proceeded to the deeper compartment of the forearm. The brachial artery was observed to divide into radial and ulnar arteries at the mid-humerus level. The radial artery replaced the brachial artery in the antecubital fossa and the ulnar artery accompanied the median nerve posterior to pronator teres into the forearm.

Conclusion: Such variations observed bilaterally have not yet been reported in the literature. Knowledge of these variations in the origin of pronator teres muscle, the absence of specific neurovascular structures as expected within the cubital fossa, and the awareness of early bifurcation and variation in their course can be very profound for physicians, as this region is often involved in the creation of arterio-venous fistulas for medical procedures, surgical treatment options for supracondylar and radial head fractures, and to differentiate median nerve compression in pronator teres syndrome versus carpal tunnel syndrome.

## Introduction

During clinical practice, physicians need to have a sound knowledge of vascular and nerve variations. Patients presenting with various clinical signs and symptoms must be thoroughly investigated with anatomic variations in mind to prevent misdiagnosis. Most nerve variations are related to their formation or their course and are frequently associated with the variability of structures that surround them. These structures most commonly include blood vessels, ligaments, and muscles. Such variations should be foremost in a physician’s mind when analyzing clinical symptoms. This will aid in accurate diagnosis, and if surgical intervention is warranted, such awareness would minimize intraoperative errors. During the dissection of 11 cadaveric specimens, an elderly female cadaver exhibited bilateral variations in the origin of pronator teres associated with neurovascular structures in the arm, elbow, and forearm. The variations in neurovascular structures involved the brachial artery and median nerve and have been reported recently [1].

The lateral border of the pronator teres muscle forms the medial border of the cubital fossa. The median nerve innervates this muscle. The function is to pronate the forearm from a supine position. This muscle originates as two heads. The ulnar head originates from the coronoid process of the ulna and the humeral head from the medial epicondyle of the humerus. Both these heads join to form the muscle belly and attach to the midshaft of the radius. In 75-80% of the population, the median nerve passes through the two heads of pronator teres [2].

Unlike the normal origin at the medial epicondyle, in this case study, the humeral head of the pronator teres muscle originated at the mid humerus level bilaterally, associated with the passage of the median nerve and ulnar artery posterior to it. This muscle was 19 cm in length bilaterally, approximately 5.5 cm longer than the average lengths of pronator teres measured bilaterally in the other cadavers. The median nerve passage showed variations bilaterally due to the abnormal origin of pronator teres most probably associated with its abnormal embryological origin. The brachial artery was observed to divide at mid humerus level into radial and ulnar arteries [1]. Knowledge of such variations can be very profound for physicians, as this region involves significance in the creation of arterio-venous fistulas, surgical treatment options for supracondylar and radial head fractures, and the differentiation and treatment of symptoms of median nerve compression in pronator teres syndrome (PTS) versus carpal tunnel syndrome (CPS).

## Materials and methods

Within the gross anatomy lab of the Geisinger Commonwealth School of Medicine campus in Scranton, Pennsylvania, dissections of cadavers were performed as part of the anatomy curriculum for first-year medical students. A total number of 11 cadavers were involved. Dissection of the upper limb in an elderly female cadaver showed variations in musculature and neurovascular structures around it. Careful dissection of the boundaries of the cubital fossa was performed by students and faculty involved, to discover that the medial boundary, which is normally formed by the pronator teres muscle, originated much higher than what is described by textbooks as normal. The adjacent neurovascular structures showed variations due to this abnormally long pronator teres muscle and these structures were also explored in detail by fine dissection. The origin and insertion of the pronator teres muscles were examined in detail for anomalies in the rest of the 11 cadavers in the lab, and lengths of these muscles were measured by a tape bilaterally for comparison. Of the 11 cadavers, 6 were males and 5 were females. The lengths of the pronator teres in the study cadaver were also measured bilaterally using a tape. The findings of the dissection regarding the pronator teres muscles and adjacent neurovascular structures are provided in the results section.

## Results

Arterial relations

In this cadaver, the brachial artery is divided into radial and ulnar arteries at the mid-humerus level. The ulnar artery traveled with the median nerve beneath the pronator teres. The radial artery traveled through the cubital fossa, replacing the brachial artery. The bicipital aponeurosis covered only the radial artery in this case, as all the other structures beneath were abnormal.

Length of pronator teres

This muscle of the flexor compartment of the forearm normally originates from the medial epicondyle of the humerus (referred to as a humeral head) and from the ulna (referred to as an ulnar head) and inserts into the id radius. The lengths of pronator teres of 11 cadavers in the lab were measured bilaterally and compared. The lengths were variable based on the height of the individuals. Some of the muscle specimens were atrophied, and some remained robust. However, the humeral heads of pronator teres from all the cadavers originated around the medial epicondyle, joined the ulnar heads, and were observed to be inserted into the mid radius. There were no neurovascular abnormalities regarding the course and distribution were noted in the other cadavers. In the cadaver demonstrating a bilateral variation in pronator teres, the lengths of this muscle were 19 cm bilaterally. This measurement was 5.5 cm longer than the average bilateral lengths of the pronator teres in other cadavers. The measurements of this muscle bilaterally in the remaining 10 cadavers are shown below in Table 1.

**Table 1:** The lengths of pronator teres in 10 other cadavers are shown here. The study specimen indicates the cadaver in which the abnormality was observed.

Cadaver numbers	Right limb (cm)	Left limb (cm)
Cadaver #1 (female)	10	10
Cadaver #2 (female)	12	12
Cadaver #3 (male)	14	13
Cadaver #4 (male)	14	14
Cadaver #5 (male)	16	15
Cadaver #6 (female)	11	10.5
Cadaver #7 (male)	14	14
Cadaver #8 (female)	13	14
Cadaver #9 (male)	15	15.5
Cadaver #10 (male)	17.5	17.5
Study specimen (female)	19	19

Due to the abnormal proximal origin of pronator teres, the median nerve traveled posterior to the muscle to the elbow and showed variation in its passage between the two heads at the apex of the cubital fossa. On the right side, the median nerve ran medial to the medial epicondyle and posterior to pronator teres at the elbow joint and coursed between the two heads as it entered the flexor compartment and innervated the muscles such as pronator teres, flexor carpi radialis (FCR), palmaris longus, flexor digitorum superficialis (FDS), flexor pollicis longus (FPL), pronator quadratus and lateral half of flexor digitorum profundus (FDP). On the left side, the median nerve ran posterior to pronator teres but ran medially to both heads at the elbow to enter the flexor compartment and innervated the muscles of the forearm mentioned above. On both sides, this nerve was accompanied by an ulnar artery from the mid-humerus level to the apex of the cubital fossa. The findings are described in the following images as upper limb findings and findings within the elbow and proximal forearm regions.

**Figure 1:**
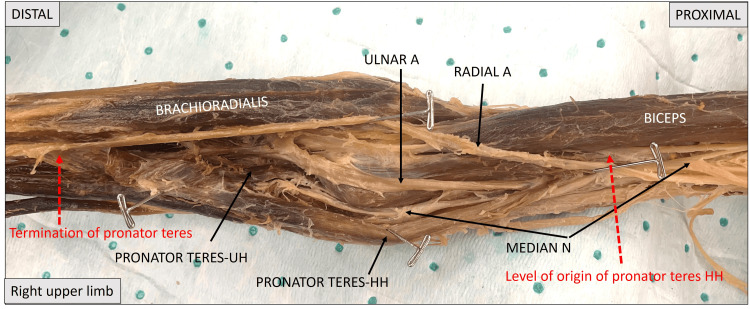
Image of the right upper limb covering the arm, elbow, and proximal forearm. The extent of pronator teres is indicated by red dotted arrows. The median nerve is observed to emerge from beneath the pronator teres to travel between its two heads. Pronator teres HH: pronator teres humeral head; pronator teres UH: pronator teres ulnar head

Figure 1 below shows a lower magnification of the right upper limb covering the mid-arm to mid-forearm level. Toward the proximal aspect, the brachial artery can be seen branching into radial and ulnar arteries. The radial artery can be seen passing through the cubital fossa. The extent of pronator teres is marked by red dotted arrows. Retraction of the pronator teres shows the median nerve and ulnar artery posterior to the muscle and entering the apex of the cubital fossa and eventually disappearing to innervate the flexors of the forearm.

**Figure 2:**
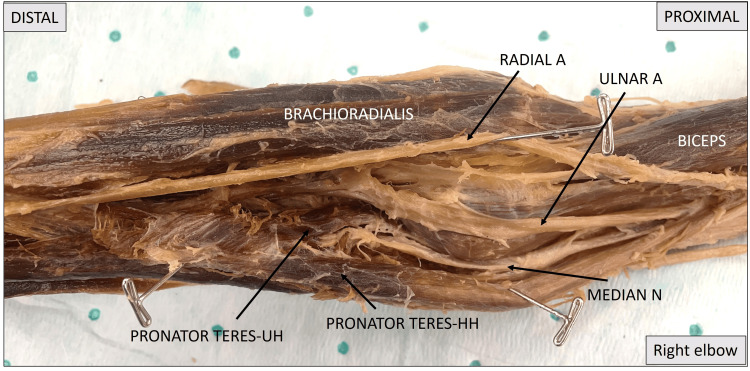
A close-up image showing the right elbow and the passage of the median nerve in relation to the pronator teres muscle. Pronator teres HH: pronator teres humeral head; pronator teres UH: pronator teres ulnar head

Figure 2 is a close-up view of the right elbow and proximal forearm area. Toward the right is the humeral end showing the biceps and toward the left is the distal end, showing the forearm. The image shows the course of the median nerve entering the elbow initially posterior and medial to the humeral head of the pronator teres muscle and then passing between the two heads to eventually enter the flexor compartment of the forearm. The radial artery can be observed to pass across the cubital fossa.

**Figure 3:**
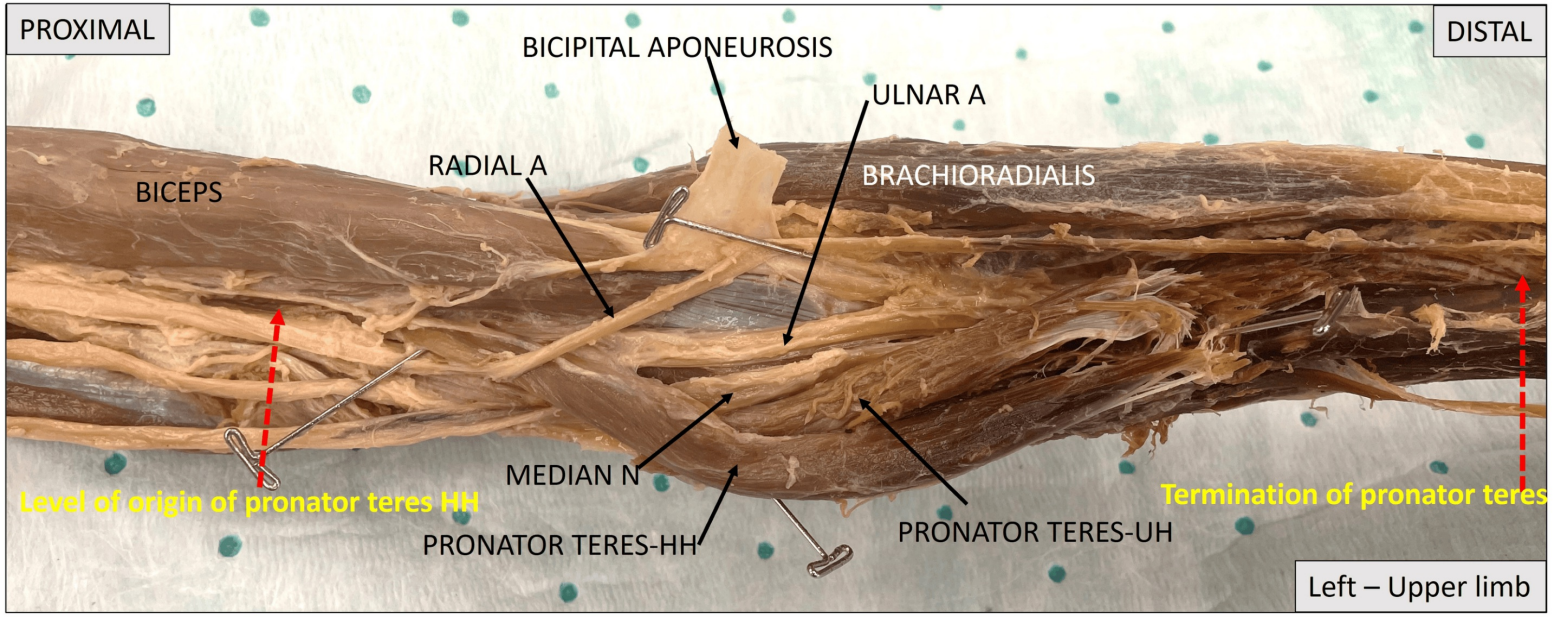
Image of the left upper limb covering the distal arm, elbow, and proximal forearm, highlighting the extent of pronator teres with red dotted arrows. Pronator teres HH: pronator teres humeral head; pronator teres UH: pronator teres ulnar head

Figure 3 shows a cadaveric image of the left upper limb, focusing on the arm, elbow, and proximal forearm. Mid-humerus to mid-forearm is covered in this image. To the left is the proximal end highlighting the biceps muscle, medial to which is the median nerve and ulnar artery, which dive beneath the pronator teres muscle at mid-humerus level. At the elbow, the bicipital aponeurosis has been cut and reflected to show the ulnar artery and median nerve emerging from beneath the humeral head of pronator teres and then passing medial to the two heads of the pronator teres and entering the flexor compartment at the apex of the cubital fossa. The radial artery can be seen coursing through the cubital fossa. The distal end of the muscle does not show the proper insertion into the radius as it was cut and reflected for exploration of this region to assess the course of the median nerve and its distribution. The site of muscle insertion is indicated by the red dotted arrows.

**Figure 4:**
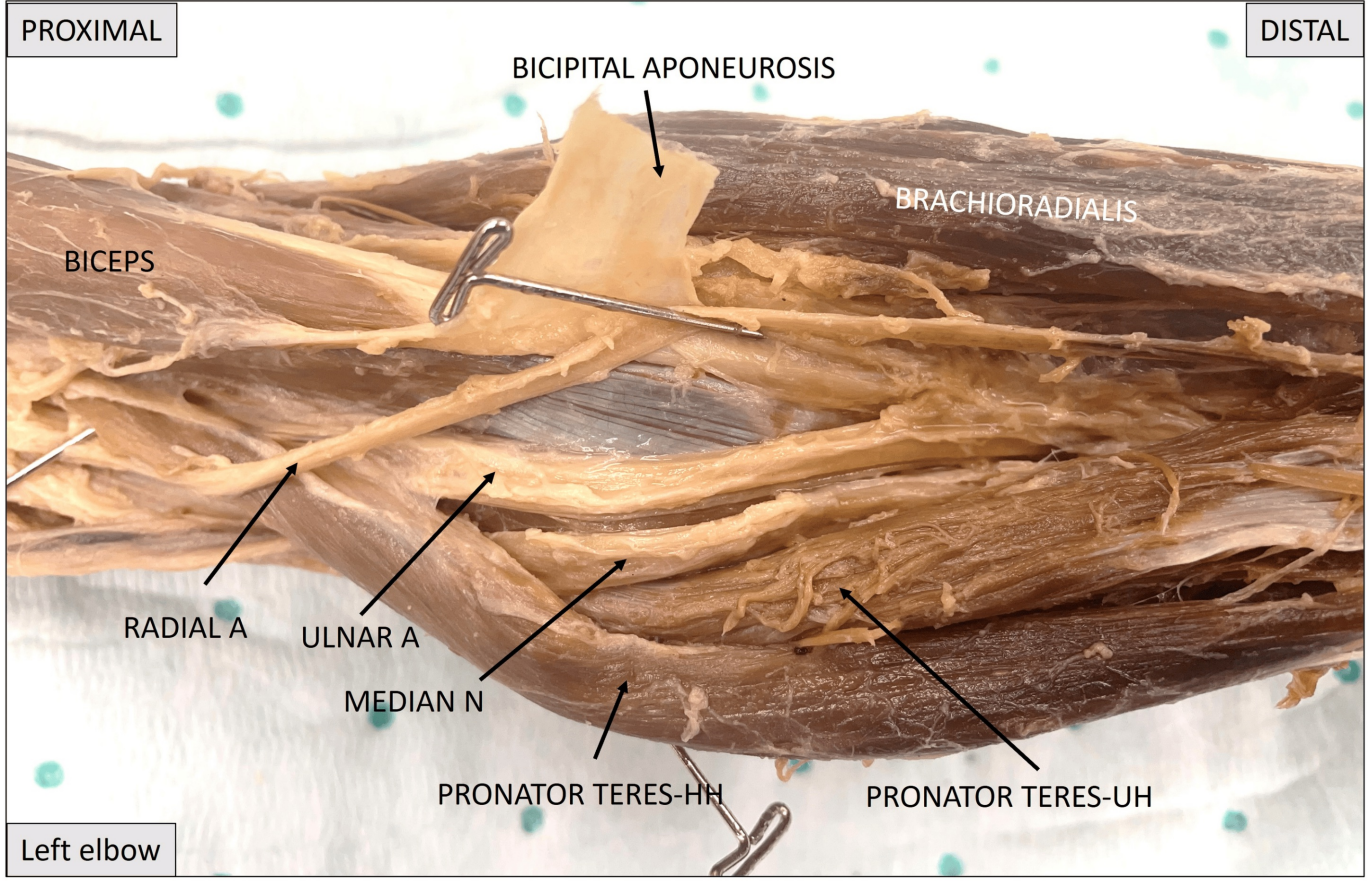
Close-up image of the left elbow showing the median nerve passing medial to both heads of pronator teres. Pronator teres HH: pronator teres humeral head; pronator teres UH: pronator teres ulnar head

Figure 4 shows the close-up view of the left elbow region. The humeral and ulnar heads of pronator teres have been mobilized medially to show the ulnar artery and median nerve as the course medial to the two heads to enter the apex of the cubital fossa. The radial artery can be seen as a component of the cubital fossa.

## Discussion

The variations from normal anatomy are important in many clinical settings. We will consider some implications for surgery and clinical differentiation between PTS and CPS. The neurovascular variations found in this cadaver were unique and were published to emphasize the clinical relevance to the specific region [1]. However, the muscular anomaly is discussed in detail here.

Regarding surgery, the abnormally high insertion of the pronator teres must be considered in the approach of any surgery to decompress the median nerve in the proximal forearm. Surgery to decompress the proximal median nerve may be indicated in individuals with high insertion of the pronator teres above the medial epicondyle of the humerus experiencing symptoms of PTS. It was found by Adler and Wolf that pronator teres with insertion at least 2 cm proximal to the medial epicondyle could have fascia that coalesces with the lacertus fibrosus in a position that can compress the median nerve [3].

Another surgical consideration is the position of the median nerve relative to both the humeral and ulnar heads of the pronator teres. In the variation seen here, the median nerve was positioned posterior to the ulnar (deep) head of the pronator teres. This could have implications during surgical decompression, as there are several potential sites of compression. Dellon and Mackinnon identified five potential sites of median nerve compression including the lacertus fibrosus, fibrous arches beneath the superficial and deep heads of the pronator teres, fibrous arch within the FDS, and accessory head of FPL (Gantzer’s muscle) [4]. As discussed, high insertion of the pronator teres above the medial epicondyle can lead to compression by the lacertus fibrosus. Similarly, positioning of the median nerve deep to the ulnar head of pronator teres could alter the relationship between the median nerve and fibrous arches of the pronator teres and FDS. Therefore, it is important to address all possible sites of compression when surgically decompressing the median nerve in the forearm [4,5]. Even with normal anatomy, releasing the deep fascia of the superficial head of pronator teres has been shown to provide symptomatic relief of PTS in up to 93% of patients [6].

One additional surgical implication of this anatomical variation is the positioning of the median nerve, brachial artery, and radial and ulnar arteries relative to the antecubital fossa. As with any surgery, identifying and protecting neurovascular structures is paramount to patients’ safety and outcomes. Surgeons must always remember that neurovascular structures may be positioned differently than expected, which can affect surgical approaches for procedures such as nerve decompression and fracture repairs.

PTS is commonly misdiagnosed as CPS, as both are associated with median nerve compression. Patients may suffer from paresthesia and pain in the median nerve distribution, namely the thumb, index, middle, and radial half of the fourth finger. Anatomically, CPS refers to median nerve compression in the carpal tunnel region, while PTS involves median nerve compression in the proximal forearm [4,5]. Despite the overlapping nature of their respective symptoms, there are some key differentiating characteristics between the two conditions. Patients with PTS often report paresthesia to the thenar eminence, while thenar atrophy is a sign of advanced CTS that signifies significant motor function loss. Patients with PTS may also experience discomfort to the proximal forearm, especially with persistent elbow flexion and forearm supination and pronation. This can be attributed to the stretching of the pronator teres and the lacertus fibrosus, leading to further compression of the median nerve [3]. Provocative maneuvers may help narrow down differential diagnoses. Tinel, Phalen, and Durkan's tests are commonly used to diagnose CTS, notably if the patient experiences pain or paresthesia to fingers innervated by the median nerve. These tests are often negative in cases of isolated PTS. Applying sustained pressure over the pronator teres for 30 seconds may trigger paresthesia in patients with PTS; no studies have been reported with positive pronator teres pressure tests in isolated CTS [7,8]. Electrodiagnostic studies are often negative or unreliable in PTS but are frequently positive in CTS. Treatment should be tailored to the patient's overall condition and symptom severity, with conservative treatment recommended for mild to moderate symptoms in both syndromes. Surgical treatment should be considered for severe symptoms or concerning diagnostic test results, such as signs of median nerve injury [9].

Fractures to the supracondylar humeral fractures, which often occur in pediatric patients, may also lead to the development of median nerve compression symptoms. During such injuries, the anterior interosseous nerve, a branch of the median nerve, is vulnerable to injury during posterolateral displacement; this may lead to weakness in the hand flexors, with difficulty in thumb abduction, and altered sensation to the palmar surface of the fingers innervated by the median nerve [10,11]. Anatomical variations to the supracondylar process may predispose patients to the development of PTS. The ligament of Struthers is an anatomical variation that exists in 1-2% of the population and is a fibrous structure that travels from the distal medial humeral diaphysis to the medial epicondyle. This ligament may compress the median nerve proximal to the medial epicondyle; it serves as a rarer scenario of median nerve compression. However, patients with the ligament of Struthers who sustain a supracondylar humeral fracture are liable to develop PTS [3]. Tumors and inflammation may also cause median nerve compression symptoms. Afshar reported a case of PTS caused by a schwannoma of median nerve origin. The tumor was located between the two heads of the pronator teres, leading to sensory loss in the radial 3.5 fingers, with swelling to the left antecubital fossa and associated pain and weakness. Upon palpation of the antecubital fossa, a hard mass was found lateral to the proximal flexor; an ultrasound of the proximal forearm revealed a soft tissue tumor adjacent to the median nerve. The patient's symptoms resolved upon complete surgical resection of the tumor, with no median nerve deficits [8]. Inflammation may also cause median nerve compression but is more likely to cause CTS rather than PTS. Local reactions like tenosynovitis and infections like histoplasmosis may trigger CTS symptoms. Inflammation may also arise from trauma-associated fractures, tumors, and cysts within the carpal tunnel, and inherent anatomical abnormalities, such as a thickened transverse carpal ligament, enlargement of the median nerve, and bony abnormalities [6].

Median nerve entrapment at the elbow, especially in athletes, presents diagnostic and treatment challenges due to its complex presentation and variable clinical symptoms compared to other median nerve compression syndromes like CPS. The median nerve may be compressed at multiple sites around the elbow and forearm, requiring thorough anatomical knowledge for accurate diagnosis and effective surgical decompression [12]. Structures near the median nerve are precisely distributed. A high humeral belly insertion of the pronator teres muscle has a potential dynamic factor for entrapment. There is a direct relationship between pronator teres muscle volume and the level at which it crosses the median nerve [13]. Thus, it is imperative for all physicians and surgeons to be aware of neurovascular and muscular anomalies within this region for efficient diagnosis and to structure precise treatment plans.

## Conclusions

Such variations in the origin of pronator teres observed bilaterally have not yet been reported in the literature. In addition to the muscular anomaly, associated neurovascular structural anomalies, as observed in this case, should always be kept in mind. Knowledge of such variations can be very profound for physicians, as this region involves clinical significance in the creation of arterio-venous fistulas, surgical treatment options for supracondylar fractures of the humerus, radial head fractures, and to differentiate median nerve compression in PTS versus CPS and treat the symptoms successfully.
